# Plasma IGF1 and 17β-Estradiol Concentrations During the Follicular Wave in Llamas

**DOI:** 10.3389/fvets.2020.555261

**Published:** 2020-10-30

**Authors:** María F. Gallelli, Carolina Bianchi, Enzo Zampini, Marcelo Aba, M. Gambarotta, Marcelo Miragaya

**Affiliations:** ^1^Consejo Nacional de Investigaciones Científicas y Técnicas, Buenos Aires, Argentina; ^2^Facultad de Ciencias Veterinarias, Instituto de Investigación y Tecnología en Reproducción Animal, Universidad de Buenos Aires, Buenos Aires, Argentina; ^3^Laboratorio de Endocrinología, Facultad de Veterinaria, Universidad Nacional del Centro de la Provincia de Buenos Aires, Tandil, Argentina; ^4^Departamento de Bioestadística, Facultad de Ciencias Veterinarias, Universidad de Buenos Aires, Buenos Aires, Argentina

**Keywords:** estradiol, folliculogenesis, follicular wave, IGF1, llama

## Abstract

The aim of this study was to characterize the temporal association between follicular waves and circulating concentrations of 17β-estradiol (E2) and IGF1 in llamas. Follicular waves could be clearly divided in three phases: growth, plateau and regression; with a mean duration of 18.8 ± 0.32 days. All follicular waves showed overlapping, so that as one dominant follicle was regressing, another one was growing. E2 plasma concentration showed a wavelike pattern, similar to that followed by the dominant follicle; reaching its maximum concentration at the end of the growth phase and decreasing at the end of the plateau phase. IGF1 also showed variations during the follicular wave. It tended to increase during the growth phase and decreased toward Days 14 and 16. IGF1 reached its maximum concentration before E2 did (5 ± 0.8 vs. 7.2 ± 0.5 days after wave emergence) and before the maximum follicular diameter was attained (10.2 ± 0.46 days after wave emergence). Both hormones started to rise again in coincidence with the development of a new follicular wave. The observed profiles allow to suggest that IGF1 could have a role on folliculogenesis and ovarian steroideogenesis in llamas, as reported for other species.

## Introduction

Llamas are induced ovulators requiring a stimulus in presence of a mature follicle to trigger the ovulatory process (1, 2). In absence of this stimulus, ovarian activity occurs in waves of follicular growth and regression, in which one follicle becomes dominant, grows to maturity and finally regresses (3, 4). Consequently, follicular waves may be divided into three phases: growth, mature and regression; with a total duration of 17–22 days, according to different authors (3, 5–7). Regression and growth of successive dominant follicles usually overlap, so that as one dominant follicle is regressing, another one is growing to dominance (7). A positive correlation between follicle size and plasma estradiol has been reported in llamas, with maximum concentrations coinciding with maximum follicle size (3, 4, 7). Thus, the follicular wave pattern and the hormonal profiles are responsible for long periods of behavioral estrous. On the contrary, plasma progesterone levels remain low during the follicular wave, since ovulation does not occur (3).

Although in the last years hormonal regulation of folicullogenesis in camelids has been studied, the mechanisms underlying the process are not yet fully elucidated (4, 8). Conversely in other species, it has been reported that folicullogenesis is regulated not only by gonadotrophins, but also by local ovarian hormones (like activin or inhibin), and other factors such as Insulin like growth factor 1 (IGF1) (9, 10). This hormone stimulates proliferation and differentiation of granulosa cells, and it would play a role in follicle selection (9, 11–13). In fact, in many domestic animals, it has been reported that IGF1 concentrations in follicular fluid are related to follicle size (11, 14). In addition, IGF1 stimulates ovarian steroideogenesis and vascularization (12, 15, 16) acting throughout its own receptor (IGF1R) which is expressed in ovarian follicles in different species [cattle: (12); ewe: (17); dog: (16)]. Recently, IGF1R was identified in ovarian follicles of alpacas (18) and llamas (unpublished data); being its expression greater in granulosa than in theca interna cells of tertiary follicles, especially in absence of a corpus luteum. This information suggests a possible effect of IGF1 on camelids ovaries.

The objective of this study was to characterize the temporal association between follicular waves and circulating concentrations of 17β-estradiol (E2) and IGF1 in llamas.

## Materials and Methods

### Animals

This study was approved by the ethics committee of the Faculty of Veterinary Sciences of Buenos Aires University (CICUAL N° 2016/27). Animals belong to the Faculty of Veterinary Sciences of Buenos Aires University (Argentina) and the experimental procedures were performed from January to August at this institution (34°36′ S, 58°22′ W, at sea level). Ten (*n*= 10) non-pregnant, non-lactating, sexually mature, clinically healthy llamas, ranging between 5 and 10 years of age and with an average body weight of 120 ± 20 kg were included in the study. All females were kept isolated from males. They were kept at grass and were fed hay bale or pellets and water *ad libitum*. They were all in good nutritional status with a mean body condition score of 3 (body condition score from 1 = thin to 5 = obese) (19).

### Follicular Activity Characterization

Animals were examined daily by rectal palpation and transrectal ultrasonography to assess ovarian status (Berger® LC 2010 plus attached to a 5.0 MHz linear-array electronic transducer, Buenos Aires, Argentina). The examination procedure was similar to that described by Bravo et al. (1). A follicular wave was defined as the simultaneous growth of a group of follicles; and a dominant follicle was defined as one that grew to at least 7 mm and whose diameter exceeded that of all other follicles in the cohort (5). The day of wave emergence (Day 0) was defined retrospectively, as the day on which the dominant follicle was first detected, at a diameter of 3–4 mm (5). The follicular wave was divided in three phases: growth, plateau and regression. The growth phase was defined as the period comprehended between the day on which the follicle emerged until it maintained its growth around the maximum diameter. The plateau phase, that followed the growth phase, was characterized by variations in follicle's diameter ≤ 0.5 mm. The regression phase was characterized by two consecutive decreasing measurements of the follicle and finished when it reached 3–4 mm at the end of the wave (5, 7). In each occasion, the diameter of each follicle was measured three times, and the averaged diameters were considered the diameter of that follicle.

### Blood Sampling

For the evaluation of plasma E2 and IGF1 concentrations, blood samples were collected by jugular venipuncture every other day. Blood samples were collected in tubes with heparin and centrifuged immediately. Plasma was stored at −20°C until IGF1 and E2 assays were performed.

### Hormone Determinations

Plasma E2 concentration was determined using a commercial RIA kit (Estradiol double antibody, KE2D, Siemens Medical Solutions Diagnostics, CA, USA) reported for use in bovine plasma (20) and validated for use with llama plasma after minor modifications (2). The sensitivity of the assay was 1.5 pg ml^−1^ and the intra-assay and the inter-assay coefficient was below 11 and 8% for concentrations between 1.5 and 48 pg ml^−1^, respectively. All samples were measured in duplicates.

IGF1 was determined by RIA as reported in cattle (21). Briefly, samples and standards were subjected to the acid-ethanol cryoprecipitation method previously described by Breier et al. (22). Concentrations of IGF-I were determined using an antibody (UB2-495) (rabbit anti-hIGF-I) provided by L. Underwood and J. J. Van Wyk and distributed by the Hormone Distribution Program of the NIDDK. Serially diluted llama plasma samples containing high IGF1 concentrations produced curves parallel to the standard curve. Assay sensitivity was 15.6 ng/ml. Intra- and interassay coefficients of variation were below 7.2 and 12.8%, respectively. All samples were measured in duplicates.

### Statistical Analysis

Hormone concentrations along the follicular wave were analyzed by repeated measures one-way ANOVA, followed by the Bonferroni test (Graph Pad 5, USA). Llamas were considered as a blocking factor. Pearson's correlation was calculated to study the relationship between IGF1 and E2. Values are expressed as mean ± SEM. Differences were considered significant when *P-*values were < 0.05 and a tendency was considered when *P* < 0.1.

## Results

### Follicular Wave Characteristics

**Figure 1:**
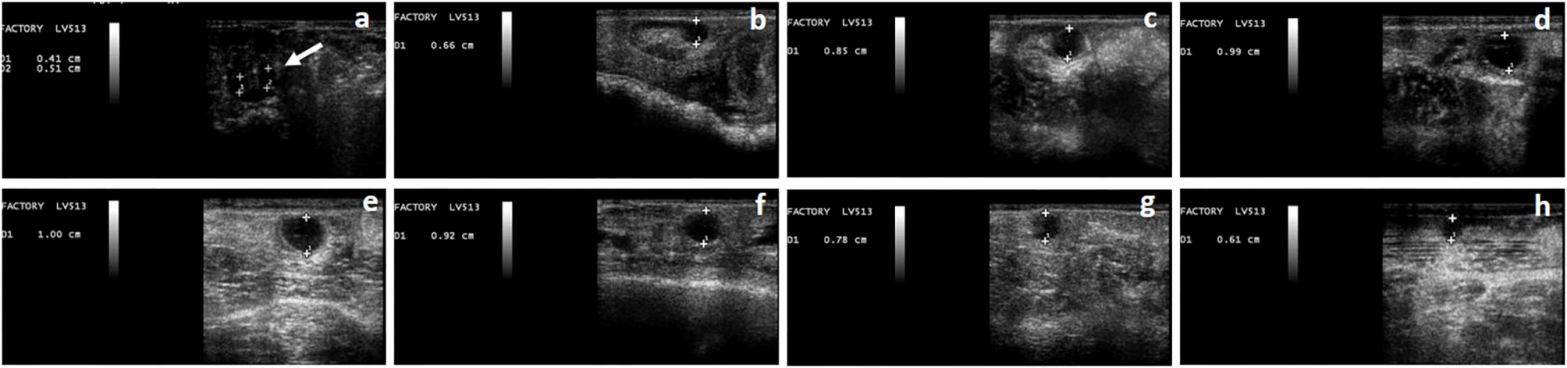
Phases of the follicular wave in a llama. The growth phase **(a–d)**, plateau phase **(d,e)**, regression phase **(f–h)**, can be observed. At the beginning of the growth phase, the simultaneous growth of a group of follicles can be observed **(a)**, one of which will become dominant (indicated by the arrow). Follicle's diameter is expressed in cm.

In all the studied animals, the follicular waves could be clearly divided in the mentioned phases (growth, plateau, and regression) (Figure 1). Mean follicular wave duration was 18.8 ± 0.32 days. The mean maximum follicular diameter was 9.9 ± 0.1 mm and it was attained at 10.2 ± 0.46 days after emergence. In all cases, the dominant follicle showed ovulatory diameter (≤ 7 mm) at day 7 after wave emergence.

The follicular growth phase averaged 9 ± 0.3 days, the plateau phase 4.2 ± 0.3 days, and the regression phase 6.7 ±0.26 days. The mean growth rate was 0.66 ± 0.03 mm/day while the mean regression rate was 0.71 ± 0.04 mm/day.

Mean diameter of the dominant follicle differed along the follicular wave (*P* < 0.001). Significant differences were detected from Day 0 until Day 6 (*P* < 0.001). Mean follicular diameter did not differ significantly later between Days 6 vs. 8, 10, 12, and 14 (*P* > 0.05). However, mean follicular diameter was greater on Day 6 than Days 16 and 18 (*P* < 0.001).

All follicular waves showed overlapping, so that as one follicle was regressing, another follicle was growing. Ovulatory follicles developed 80% times in the left ovary and 20%, in the right ovary. The sequential development of ovulatory follicles alternated between ovaries in 55% of cases. No ultrasonographical evidences of ovulation were recorded during the study.

### Plasma E2 and IGF1 Concentrations During the Follicular Wave

#### Estradiol

**Figure 2:**
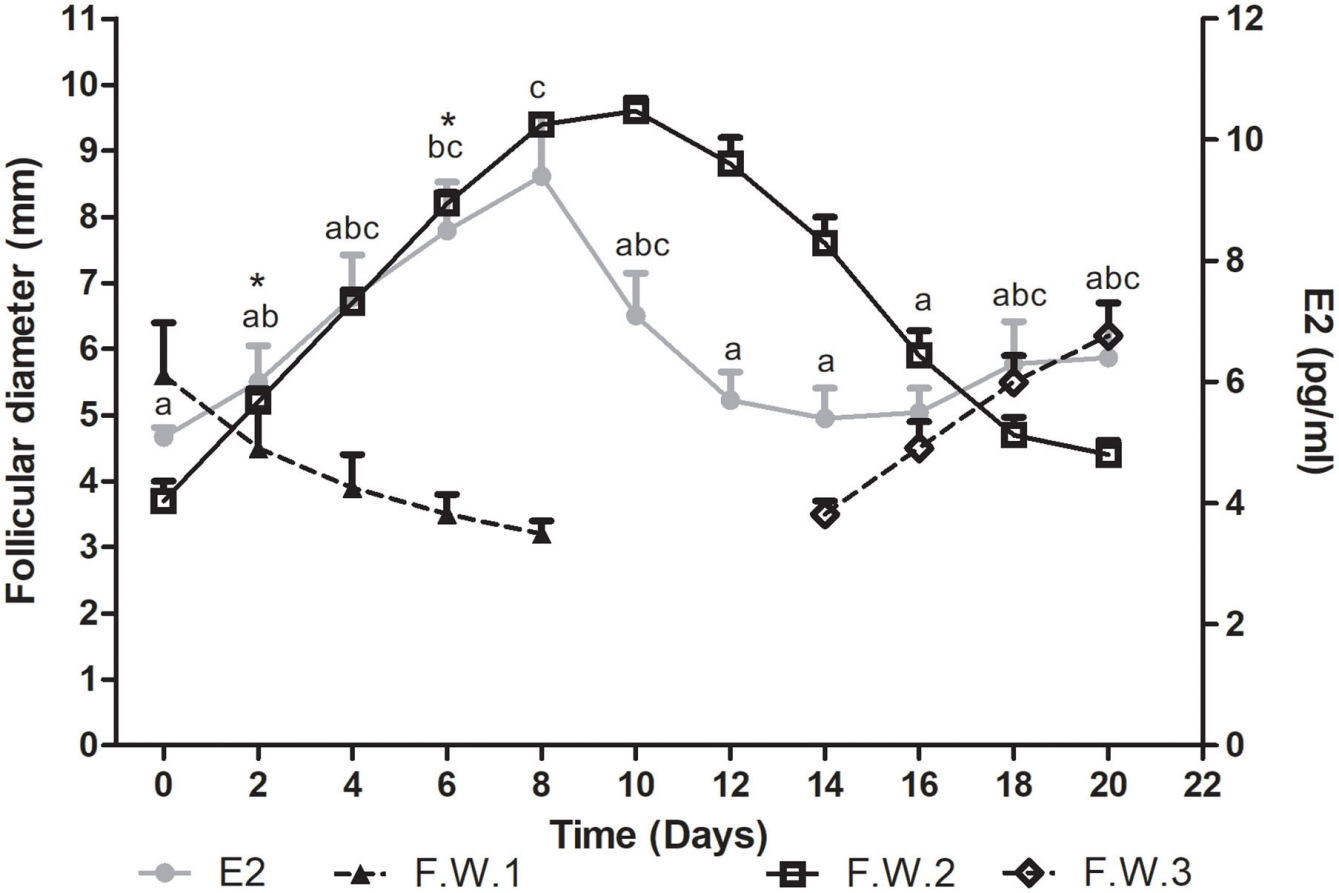
Mean follicular diameter (mm) and plasma E2 concentration (pg/ml) during the follicular wave of llamas. Follicular wave 2 (F.W.2) emerged at Day 0 and the dominant follicle went through its different phases (growth, plateau, and regression) while the dominant follicle of the follicular wave 1 (F.W.1) (previous follicular wave) was regressing. E2 plasma concentration showed a wavelike pattern similar to the one described by the dominant follicle of F.W.2. At the end of the study, as a new follicular wave initiated (F.W.3), E2 plasma concentration started to rise again. Values are expressed as mean ± SEM. Different letters indicate E2 concentrations significantly different (*P* < 0.05). * Indicates that E2 concentration showed a tendency to be greater at Days 6 vs. 2 (*P* < 0.1).

Plasma estradiol concentration showed significant differences during the follicular wave (*P* = 0.0007). This hormone showed a wavelike pattern similar to that described by the dominant follicle. Plasma E2 concentration increased during the growth phase of the follicular wave, reached its maximum concentration at the end of this phase and then started to decrease at the end of the plateau phase. The E2 mean wave length was of 12.9 ± 0.7 days, being shorter than the mean duration of the follicular wave. Thus, E2 started to decline before the structural regression of the follicle. As all follicular waves showed overlapping, plasma E2 concentration started to increase during the regression phase of the previous wave in association with the growth phase of the successive follicular wave (Figure 2).

The mean maximum E2 concentration reached was 38.2 ± 4.5 pmol/l (10.34 ± 1.2 pg/ml) and it was attained at 7.2 ± 0.5 days after wave emergence. Mean E2 concentration was significantly greater on Days 6 vs. 0, 12, 14, and 16 (*P* < 0.05) and showed a tendency to be greater on Days 6 vs. 2 (*P* < 0.1). It also was significantly greater on Days 8 vs. 0, 2, 12, 14, and 16 (*P* < 0.05) (Figure 2). The mean diameter of the dominant follicle at the day of mean maximum E2 concentration was 8.7 ± 0.3 mm.

#### IGF1

**Figure 3:**
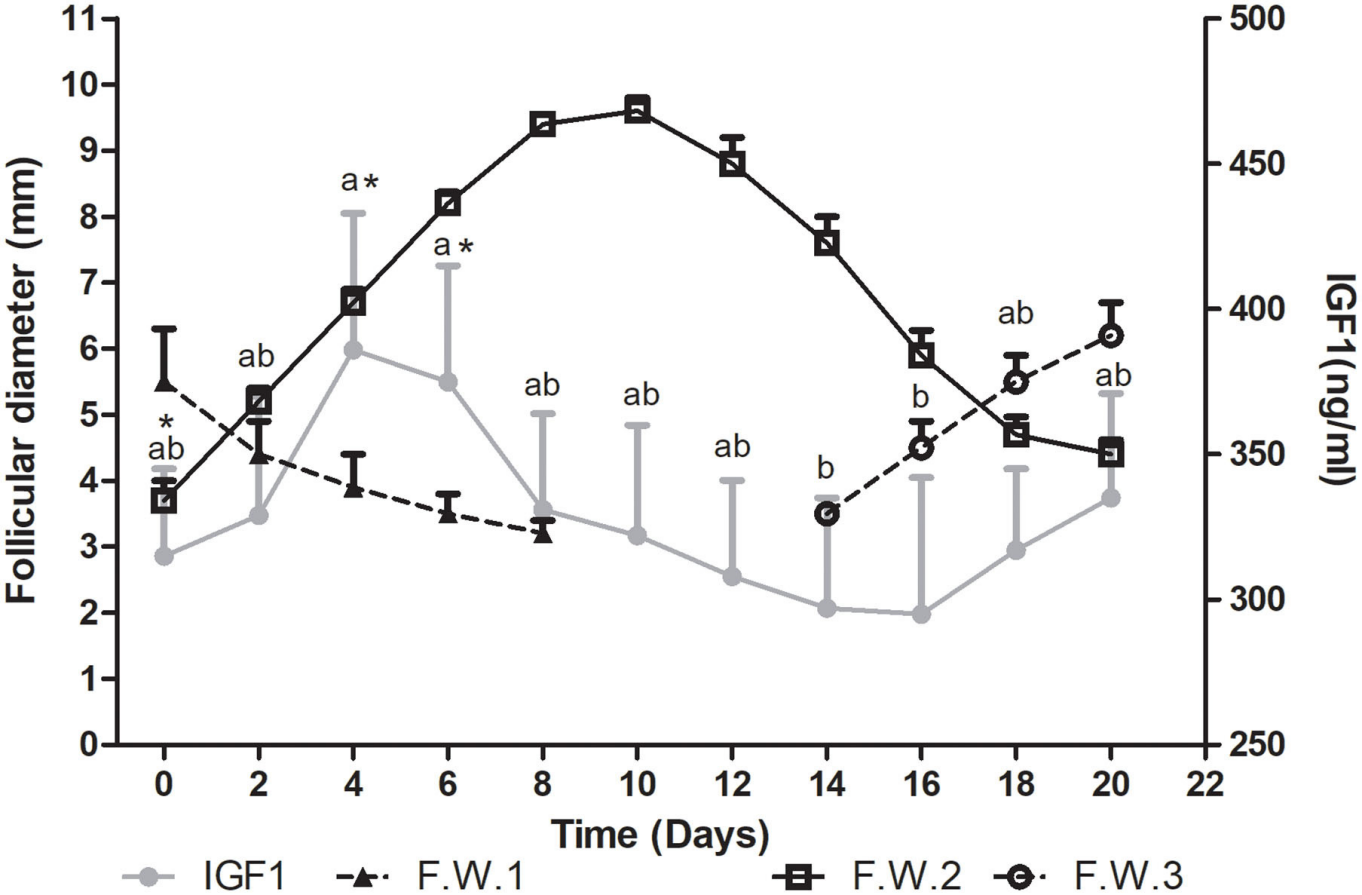
Mean follicular diameter (mm) and mean plasma IGF1 concentration (ng/ml) during the follicular wave of llamas. Follicular wave 2 (F.W.2) emerged at Day 0 and the dominant follicle went through its different phases (growth, plateau, and regression) while the dominant follicle of the follicular wave 1 (F.W.1) (previous follicular wave) was regressing. IGF1 plasma concentration reached its greatest concentration during the growth phase. At the end of the study, as a new follicular wave initiated (F.W.3), IGF1 plasma concentration started to rise again. Values are expressed as mean ± SEM. Different letters indicate IGF1 concentrations significantly different (*P* < 0.05). * Indicates that IGF1 concentration showed a tendency to be greater at Days 4 and 6 vs. 0 (*P* < 0.1).

Plasma IGF1 concentration showed differences during the follicular wave (*P*= 0.0004). Mean IGF1 plasma concentration tended to increase from Day 0 toward Days 4 and 6 (*P* < 0.1). Then it started to decrease, being significantly lower on Days 14 and 16 vs. 4 and 6 (*P* < 0.05) (Figure 3).

During the regression phase and simultaneously with the growth phase of the successive follicular wave, plasma IGF1 concentration started to increase (Figure 3).

The mean maximum IGF1 concentration was 460 ± 52 ng/ml and it was attained at 5 ± 0.8 days after wave emergence. Therefore, the mean maximum IGF1 concentration was attained during the growth phase, before the day when the mean maximum follicular diameter was reached (10.2 ± 0.46 days after wave emergence) and before the day of maximum plasma E2 concentration (7.2 ± 0.5 days). The mean diameter of the dominant follicle at the day of mean maximum IGF1 concentration was 7 ± 0.7 mm and mean plasma E2 concentration was 7.5 ±0.6. The mean lowest IGF1 concentration was 250 ± 39 ng/ml and it was attained at 11± 1.3 days after wave emergence, previous to the structural regression of the dominant follicle (regression phase) (Figure 3).

### Correlation Analysis

There was correlation between IGF1 and E2 plasma concentration (*r*^2^ = 0.31, *P* = 0.008).

## Discussion

In the present study, a temporal association between follicular waves and circulating concentrations of E2 and IGF1 in llamas has been demonstrated.

In agreement with previous studies, the development of the dominant follicle showed a wavelike pattern that could be divided into three phases (growth, plateau, and regression) (3, 7). The mean length of the follicular waves and their phases was shorter than that previously reported by some authors (3, 7) but similar to that described by others (5, 6). However, it must be considered that follicular wave length is highly variable, lasting from 17 to 25 days, and closely related with the maximum diameter reached by the dominant follicle (7). The phenomenon of overlapping of follicular waves has been observed in all cases. Likewise, in a study performed in llamas it has been reported that 100% of follicular waves were superimposed on the preceding ones (7). On the contrary, Bravo et al. (1) referred absence of overlapping in some follicular waves, in coincidence with basal E2 concentration. The underlying cause of the differences between all the mentioned studies is not clear, but it might be related to environmental or genetic factors that could have an impact on follicular activity.

Plasma E2 concentration showed a wavelike pattern, resembling that described by the dominant follicle, as previously reported in llamas (1, 3, 7). This hormone concentration increased during the growth phase, reaching its maximum at the end of this phase, and then decreased when the plateau phase was finishing. Also, E2 mean wave length was shorter than the mean duration of the follicular wave. Therefore, the structural regression of the dominant follicle was preceded by the decline in E2 concentration, so that the dominant follicle function was affected before ultrasonagraphic changes were observed. These results are consistent with those described by Cavilla et al. (7), who observed an increase of E2 concentration in coincidence with follicular wave emergence and a decrease during the plateau phase. Likewise, in cows and sheep, E2 reached its maximum concentration at the end of the growth phase and started to decrease during the static phase (23, 24). Thus, it has been proposed that the decline in E2 concentration during this phase could have a role in the development of the subsequent follicular wave (24). In the present study, during the regression phase, a new follicular wave started to develop, leading to a new increase in plasma E2 concentration.

Plasma IGF1 concentration showed variations during the follicular wave; being the first time that this factor was evaluated in llamas. IGF1 concentration tended to increase during the growth phase and then decreased toward Days 14 and 16. As the new follicular wave emerged, IGF1 started to rise again. To the authors knowledge, there are no reports regarding the effect of IGF1 on ovarian function in llamas. However, in other species it has been proposed that this factor would promote folliculogenesis, stimulating granulosa cells proliferation and follicular steroideogenesis [sheep: (25); pig: (26); cattle: (27, 28); goat: (29); horse: (13)]. Considering the observed IGF1 profile, it would be possible that in llamas this factor might be involved in the stimulation of follicular development, as in other species. Besides, variations in IGF1 plasma concentration during the follicular phase of the estrous cycle have been observed in domestic species [sheep: (25, 30); goat: (29); cattle: (28)]. These authors reported that plasma IGF1 concentration increased in association to estrous, in coincidence with maximum plasma E2 concentration. Moreover, *in vitro* studies have shown that IGF1 stimulates E2 production by granulosa cells in a dose dependent manner (31–33). Conversely, it has also been proposed that E2 would stimulate IGF1 production (25, 28). In the present study, a positive correlation between IGF1 and E2 has been observed. Opposite to what has been reported in other species, mean IGF1 greatest concentration was not attained simultaneously to E2 peak, reaching its maximum concentration before E2 did (5 ± 0.8 vs. 7.2 ± 0.5 days after wave emergence). These results might suggest that in llamas IGF1 would stimulate follicular E2 production, although an effect of E2 on IGF1 should not be discarded. Although correlation between E2 and IGF1 was observed, the association between these variables was not strong. This might be explained by the fact that ovarian function is regulated by multiple factors, including gonadotropins (34). In other species, it has been proposed that IGF1 might regulate follicular activity directly and/or by increasing follicle's sensitivity to gonadotropins action (11, 12, 27). The exact mechanisms by which IGF1 is involved in ovarian function regulation in camelids remains to be studied.

In conclusion, in llamas, E2 showed a wavelike pattern while IGF1 tended to increase toward Days 4 and 6 and then decreased; reaching its greatest values before E2 and the diameter of the dominant follicle did. This suggests that IGF1 might have a role in ovarian folliculogenesis and steroideogenesis. Further research is needed to elucidate the mechanism of action of IGF1 on ovarian tissue in this species.

## Data Availability Statement

The raw data supporting the conclusions of this article will be made available by the authors, without undue reservation.

## Ethics Statement

The animal study was reviewed and approved by Comité Institucional de Cuidado y Uso de Animales de Experimentación, Facultad de Ciencias Veterinarias, Universidad de Buenos Aires.

## Author Contributions

MGal designed and performed the study and wrote the manuscript. CB collaborated in the study design, performed E2 determinations, and revised the manuscript. EZ collaborated in field performance of the study. MGam performed statistical analysis and revised the manuscript. MA revised and edited the manuscript. MM collaborated in the study design and revised and edited the manuscript. All authors contributed to the article and approved the submitted version.

## Conflict of Interest

The authors declare that the research was conducted in the absence of any commercial or financial relationships that could be construed as a potential conflict of interest. The reviewer SA declared a shared affiliation with several of the authors MGal and CB to the handling editor at the time of review.
